# Correlation between histologic chorioamnionitis and severe retinopathy of prematurity

**DOI:** 10.1038/s41390-025-04093-y

**Published:** 2025-05-16

**Authors:** Young Mi Yoon, Seung Han Shin, Chan-wook Park, Ee-Kyung Kim, Han-Suk Kim

**Affiliations:** 1 Department of Pediatrics, Chungnam National University Sejong Hospital, Sejong, Korea; 2 Department of Pediatrics, Seoul National University College of Medicine, Seoul National University Children’s Hospital, Seoul, Korea; 3 Department of Obstetrics & Gynecology, Seoul National University College of Medicine, Seoul National University Hospital, Seoul, Korea

## Abstract

**Background:**

Retinopathy of prematurity (ROP) is influenced by factors, including gestational age (GA), oxygen exposure, and chorioamnionitis. However, the association between histologic chorioamnionitis (HCA) and ROP remains controversial. This study aimed to investigate the association between HCA and severe ROP.

**Methods:**

This retrospective cohort study utilized data from the National Korean Neonatal Network registry, focusing on infants with birthweights < 1500 g and GA < 32 weeks. Univariate and multivariate logistic regression analyses assessed the association between HCA and severe ROP. Sub-cohort analyses were performed to evaluate the effect of HCA on severe ROP across different GA groups.

**Results:**

Infants in the HCA cohort had lower GA and birth weights, with a higher prevalence of any-stage and severe ROP compared to those in the without-HCA cohort. However, multivariate logistic regression showed an inverse association between HCA and severe ROP. Sub-cohort analyses revealed that HCA was associated with an increased risk of severe ROP in infants born at 26–28 and 28–31 weeks, while no significant association was observed in infants born at 23–25 weeks.

**Conclusions:**

HCA may reduce the risk of severe ROP, suggesting that intrauterine inflammation could play a protective role. Further research is needed to elucidate underlying mechanisms.

**Impact:**

- Retinopathy of prematurity (ROP) is influenced by numerous perinatal and postnatal factors, including low gestational age, oxygen exposure, and chorioamnionitis. However, the association between chorioamnionitis and ROP remains controversial.
- Our study showed that histologic chorioamnionitis (HCA) was negatively correlated with severe ROP in preterm infants, even after adjusting for confounding factors such as gestational age and birth weight.
- These findings suggest that HCA may have a protective effect against severe ROP, potentially mediated by inflammatory markers.

## Introduction

Retinopathy of prematurity (ROP) is characterized by abnormal retinal blood vessel development due to incomplete vascularization of the retinal tissue and primarily affects premature infants.^1^ This condition leads to visual impairment, blindness, and adverse neurodevelopmental outcomes in preterm infants.^2–5^ With improved survival rates among premature infants, the incidence of ROP in the United States has risen from 4.4% to 8.1% over the past two decades.^6,7^ Prematurity and postnatal oxygen exposure play crucial roles in ROP development.^8–11^ Several other prenatal and postnatal factors, including histologic chorioamnionitis (HCA), respiratory distress syndrome (RDS), bronchopulmonary dysplasia (BPD), necrotizing enterocolitis (NEC), and sepsis, have also been associated with ROP.^8,11–13^

Most studies investigating the association between ROP and HCA have reported a positive correlation.^3,5,11–16^ Dammann et al. proposed a multiple-hit hypothesis, suggesting that intrauterine inflammation acts as the “first hit” against premature fetuses, while subsequent neonatal infections serve as “multiple hits”, further exacerbating the risk of ROP in preterm infants.^12^ Chen et al. observed that although placental bacteria or inflammation are not independently associated with ROP, their combination is significantly associated with an increased risk of ROP in zone I.^13^

In contrast, some studies have reported that HCA is associated with a decreased risk of ROP.^17–19^ Park et al. observed that the progression of acute HCA in infants without fetal growth restriction serves as a protective factor against ROP.^17^ A retrospective cohort study showed that HCA is associated with a reduced odds ratio of severe ROP after adjusting for gestational age (GA), birthweight, and sex.^18^ However, these studies merely compared the incidence of ROP among the study cohorts.

Given that ROP primarily affects premature infants and that HCA is more frequently observed in this population, the correlation between these two conditions should be interpreted with caution. Stratification or adjustment for GA in the analysis of HCA and ROP is essential to clarify this relationship. Therefore, this study aimed to elucidate the association between HCA and ROP using a national registry of very low birth weight infants (VLBWIs), while controlling for key perinatal and neonatal factors that are related to developmental outcomes.

## Methods

### Study population

This cohort study utilized the Korean Neonatal Network (KNN) database, a national registry established in 2013, that systematically collects standardized data on the management, morbidities, and mortalities of VLBWIs using electronic case report forms.

The study included infants registered in the KNN database born between January 2013 and June 2023 with birthweights < 1500 g and GA < 32 weeks. Exclusion criteria were as follows: 1) infants with congenital anomalies, 2) infants without HCA data, and 3) infants without a diagnosis or grading of ROP.

HCA was defined as the presence of acute inflammatory changes, including polymorphonuclear leukocyte infiltration, observed in any segment of the amnion, chorion–decidua, umbilical cord, or chorionic plate. This diagnosis was confirmed through histopathological examination of the placenta, performed by pathologists at each participating institution. The study population was divided into two cohorts: those with HCA and those without.

Certified ophthalmologists at the participating KNN-registered hospitals conducted ophthalmic assessments and administered treatments. ROP treatment included surgical interventions, such as laser photocoagulation and intravitreal injections of anti-vascular endothelial growth factor (VEGF) agents. ROP was classified according to the criteria established by the International Committee for the Classification of Retinopathy of Prematurity. Severe ROP was defined as stage 3 or higher or any ROP requiring treatment.^20^ The KNN database does not explicitly record ROP regression or progression; however, we estimated the rate of disease progression based on the proportion of infants who developed severe ROP among those diagnosed with any ROP.

Small for gestational age (SGA) was defined as a birthweight below the 10th percentile on the Fenton growth chart.^21^ Data from the KNN database were used to classify prenatal steroid administration within 7 days before delivery into three categories: no prenatal steroids, incomplete prenatal steroids, and complete prenatal steroids (including both betamethasone and dexamethasone). RDS was diagnosed based on the presence of clinical respiratory distress, characteristic radiographic findings, and the need for either invasive or noninvasive mechanical ventilation. NEC was defined as the presence of two or more stages according to the modified Bell’s criteria.^22^ High-grade intraventricular hemorrhage (IVH) was defined as three or more stages according to Papile’s criteria.^23^ Sepsis was defined as a positive bacterial or fungal culture obtained from the infant’s blood, accompanied by clinical signs of infection or treatment with appropriate antibiotics for 5 or more days.^24^ Moderate-to-severe BPD was defined as dependence on supplemental oxygen or positive pressure support at 36 weeks postmenstrual age according to the National Institutes of Health Workshop severity-based diagnostic criteria.^25^

### Statistical analyses

Univariate and multivariate logistic regression analyses were performed to assess the severity of ROP. The analyses controlled for established confounding variables known to influence the risk of ROP, including GA, sex, SGA, moderate-to-severe BPD, NEC, sepsis, and duration of invasive ventilation.^10,11^ Given the critical role of GA in ROP development, sub-cohort analyses were conducted for infants with GAs of 23–25, 26–28, and 29–31 weeks to calculate the adjusted odds ratios for severe ROP. Data are presented as numbers (%) or means ± standard deviations. All statistical analyses were conducted using STATA, version 18.0 (Stata Corp, College Station, TX**)**, with statistical significance set at *p* < 0.05.

## Results

**Fig. 1:**
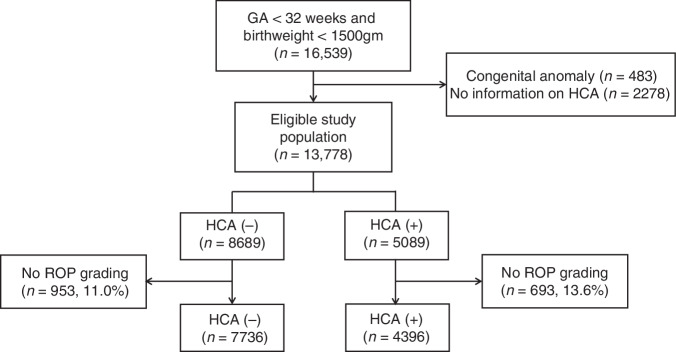
Flow chart of study population.

During the study period, 16,539 infants were born with birthweights < 1500 g and GAs < 32 weeks (Fig. 1). After excluding infants with congenital anomalies and those lacking HCA data, 13,778 infants were eligible for the study. Following further exclusions of infants with incomplete data on ROP diagnosis and grading, the final cohorts included 7736 infants in the without-HCA group and 4396 infants in the HCA group.

The GA (28.7 vs. 27.6 weeks, *p* < 0.001) and birthweights (1084.6 vs. 1045.9 g*, p* < 0.001) were significantly lower in the HCA cohort than in the without-HCA cohort (Table 1). SGA status, cesarean deliveries, and multiple births were more prevalent in the without-HCA cohort, while oligohydramnios, prenatal steroid use, and preterm premature rupture of membrane were more common in the HCA cohort.

**Table 1:** Perinatal characteristics of the study population

	Without-HCA (*n* = 7736)	HCA (n = 4396)	*p-*value
Gestational age (week)	28.7 ± 2	27.6±2.1	<0.001
Birthweight (gram)	1084.6 ± 265.3	1045.9±264.8	<0.001
Birthweight (z-score)	−0.3 ± 0.8	0.1±0.8	<0.001
SGA	1004 (13)	213 (4.9)	<0.001
Cesarean section	6597 (85.3)	3064 (69.7)	<0.001
Female	3823 (49.4)	2166 (49.3)	0.895
Apgar score 1 min	4.8 ± 2	4.6 ± 2	<0.001
Apgar score 5 min	7 ± 1.7	6.8 ± 1.8	<0.001
Oligohydramnios	852 (11.8)	716 (17.3)	<0.001
Prenatal steroid			0.020
No prenatal steroid	964 (12.6)	483 (11.1)	
Incomplete	2939 (38.4)	1641 (37.8)	
Complete	3747 (49)	2219 (51.1)	
Multiple birth	3136 (40.5)	1297 (29.5)	<0.001
PPROM	2259 (29.3)	2405 (55)	<0.001

Values are expressed as *N* (%) or means ± standard deviations (SDs).

*HCA* histologic chorioamnionitis, *SGA* small for gestational age, *PPROM* preterm premature rupture of membrane.

Neonatal morbidities, such as RDS, NEC, high-grade IVH, sepsis, and moderate to severe BPD, were more prevalent in the HCA cohort (Table 2). Additionally, any ROP (34.4% vs. 44.6%, *p* < 0.001), treated ROP (25.2% vs. 33.6%, *p* < 0.001), and severe ROP (14.8% vs. 21.4%, *p* < 0.001) were more frequent in the HCA cohort than in the without-HCA cohort. ROP progression occurred more frequently in the HCA cohort than in the without-HCA cohort (42.9% vs. 47.9%, *p* < 0.001). Regarding specific treatment modalities, anti-VEGF therapy alone was administered to 18.9% of the HCA cohort compared with 13.6% of the without-HCA cohort. Laser therapy alone was used in 8.3% of the HCA cohort and 7.3% of the without-HCA cohort. Additionally, 6.4% of infants in the HCA cohort received both anti-VEGF and laser therapy, whereas 4.3% of infants in the without-HCA cohort received the combination.

**Table 2:** Neonatal morbidities of the study population

	Without-HCA (*n* = 7736)	HCA (*n* = 4396)	*p*-value
RDS	6480 (83.8)	3760 (85.5)	0.010
NEC	473 (6.1)	279 (6.4)	0.611
IVH ≥ Gr 3	3090 (40)	2032 (46.2)	<0.001
Sepsis	1435 (18.6)	968 (22)	<0.001
Moderate to severe BPD	2673 (35.1)	1782 (41.3)	<0.001
Any ROP	2661 (34.4)	1961 (44.6)	<0.001
Treated ROP	911 (25.2)	810 (33.6)	<0.001
anti-VEGF only	492 (13.6)	456 (18.9)	
laser only	263 (7.3)	200 (8.3)	
Both	156 (4.3)	154 (6.4)	
Severe ROP	1142 (14.8)	939 (21.4)	<0.001
Duration of invasive ventilation (days)	14.7 ± 25.5	20.6 ± 33.4	<0.001
Duration of non-invasive ventilation (days)	24.4 ± 21.5	28.6 ± 23.6	<0.001
Duration of supplementary oxygen (days)	7.5 ± 12.7	9.3 ± 14.7	<0.001
Hospital days	76.7 ± 37.2	87.4 ± 43.3	<0.001
Death	124 (1.6)	95 (2.2)	0.028

Values are expressed as N (%) or means ± standards (SDs).

*HCA* histologic chorioamnionitis, *RDS* respiratory distress syndrome, *NEC* necrotizing enterocolitis, *IVH* intraventricular hemorrhage, *BPD* bronchopulmonary dysplasia, *ROP* retinopathy of prematurity, *VEGF* vascular endothelial growth factor.

Mortality was higher in the HCA cohort than in the without-HCA cohort (1.6% vs. 2.2%, *p* = 0.028). The duration of invasive ventilation (14.7 ± 25.5 vs. 20.6 ± 33.4 days, *p* < 0.001) and hospital stays (76.7 ± 37.2 vs. 87.4 ± 43.3 days, *p* < 0.001) were also longer in the HCA cohort. In an analysis of ventilation duration, infants with ROP required longer periods of invasive ventilation, non-invasive ventilation, and supplementary oxygen than those without ROP in both the HCA and without-HCA cohorts (Supplementary Table 1).

Univariate logistic regression analysis revealed that GA was inversely correlated with severe ROP (Table 3). SGA status and HCA were positively associated with severe ROP. Neonatal morbidities, including RDS, moderate to severe BPD, sepsis, and NEC, were also positively correlated with severe ROP. Multivariate logistic regression analysis showed that after adjusting for lower GA, SGA, RDS, moderate-to-severe BPD, sepsis, NEC, and duration of invasive ventilation, HCA was inversely correlated with severe ROP (adjusted odds ratio [aOR], 0.816; 95% confidence interval [CI], 0.721–0.923).

**Table 3:** Univariate and multivariate logistic regression analyses for severe retinopathy of prematurity

	OR	95% CI	Adjusted OR^a^	95% CI
GA (week)	0.456	0.441–0.472	0.476	0.458–0.494
SGA	1.203	1.036–1.398	2.090	1.714–2.550
HCA	1.568	1.425–1.726	0.816	0.721–0.923
RDS	6.669	5.201–8.551	1.331	1.002–1.767
Moderate to Severe BPD	6.118	5.501–6.804	2.111	1.859–2.398
Sepsis	2.846	2.565–3.157	1.216	1.067–1.387
NEC	2.828	2.414–3.312	1.231	1.005–1.509

*OR* odds ratio, *CI* confidence interval, *GA* gestational age, *SGA* small for gestational age, *HCA* histologic chorioamnionitis, *RDS* respiratory distress syndrome, *BPD* bronchopulmonary dysplasia, *NEC* necrotizing enterocolitis.

^a^Adjusted for GA, SGA, HCA, female sex, RDS, moderate to severe BPD, sepsis, NEC and duration of invasive ventilation.

When classified by GA into 23–25, 26–28, and 29–31 weeks, the prevalence of HCA was highest in the most immature group (55.7% for GA 23–25 weeks) and decreased with advancing GA (40.1% for GA 26–28 weeks, 25.7% for GA 28–31 weeks, *p* < 0.001) (Supplementary Table 2). Infants born at 23–25 weeks had the highest rates of neonatal morbidity and mortality. Similarly, severe ROP was most prevalent in the most immature group (59.4% for GA 23–25 weeks, 16.8% for GA 26–28 weeks, and 2.5% for GA 28–31 weeks). Due to a ceiling effect associated with lower gestational ages, the impact of HCA on the occurrence of severe ROP may be underestimated or attenuated. In extremely preterm infants (23–25 weeks), the overall risk for ROP was so high that the effects of other factors, such as HCA, became attenuated. However, in infants with relatively higher gestational ages (26–31 weeks), where the intrinsic risk of ROP was lower, the influence of HCA on the development of ROP was more apparent.

**Fig. 2:**
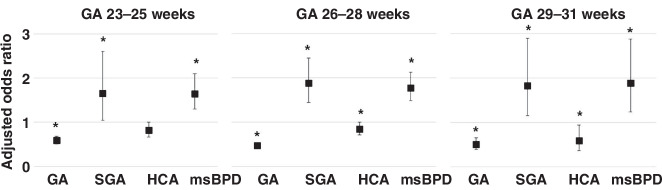
Multivariate logistic regression analysis for severe retinopathy of prematurity in the sub-cohorts of the study population. Effect of GA, SGA, HCA and moderate-to-severe BPD on severe ROP among different gestational age group.

A subcohort analysis of multivariate logistic regression for severe ROP showed that lower GA, SGA status, and moderate-to-severe BPD were positively correlated with severe ROP across all sub-cohorts (Fig. 2). HCA was negatively associated with severe ROP in the sub-cohorts of infants born at 26–28 weeks (aOR, 0.845; 95% CI, 0.714–0.999) and 29–31 weeks GA (aOR, 0.585; 95% CI, 0.364–0.942). However, no significant correlation was found in infants born at 23–25 weeks (aOR, 0.818; 95% CI, 0.667–1.004).

## Discussion

ROP is a multifactorial disease influenced by various conditions, commonly occurring in premature infants, as is HCA.^8,11–13^ Given this overlap, adjusting for risk factors, particularly GA, is essential to better understand the correlation between HCA and ROP. In this study population from the national KNN registry, severe ROP was observed more frequently in infants with HCA. However, after adjusting for confounding factors, we found a negative correlation between HCA and severe ROP in preterm infants.

Previous studies have reported a higher incidence of ROP in preterm infants with HCA. However, these studies focused only on the incidence of ROP among different study cohorts.^13,26–28^ Additionally, a few studies that adjusted for GA with or without birth weight have shown that HCA does not increase the risk of ROP.^14,29^ Interestingly, two systematic reviews also found an association between ROP and HCA; however, this association disappeared after adjusting for GA, with or without birth weight.^8,30^

In contrast, more recent studies, including those involving homogenous populations without fetal growth restrictions^17^ or larger sample sizes,^18^ have revealed that HCA is negatively correlated with ROP development in preterm infants. Although a few studies have reported an increased risk of ROP due to HCA, even after adjusting for GA with or without other factors, the study populations were not large enough to validate the correlation between HCA and ROP.^5,12^

Sub-cohort analyses were conducted to examine whether the effect of HCA on severe ROP varies with levels of prematurity. The influence of HCA on severe ROP was attenuated in the more prematurely born sub-cohort (23–25 weeks and 26–28 weeks of gestation). Since prematurity is a significant predictor of severe ROP, the impact of HCA on severe ROP may be less pronounced in more premature infants.

One hypothesis is that the potential protective effect of HCA against ROP may be associated with inflammatory markers.^19^ Intra-amniotic inflammation may reduce the intact form of insulin-like growth factor-binding protein 1 (IGFBP-1), leading to an increase in insulin-like growth factor 1 (IGF-1) levels, a crucial factor in retinal vascular development.^17,31^ Because IGFBP-1 inhibits free IGF-1 and reduces IGF signaling, its decrease in HCA could increase IGF-1 levels, potentially supporting normal retinal vessel development.^31–33^ However, as these findings are based on associations rather than direct evidence for causality, further studies are necessary to clarify the underlying mechanisms.

This study has certain limitations. First, 3.8% of the registered population lacked HCA data, and 11.9% lacked ROP diagnosis and grading data. Severe ROP was more common in infants with HCA-related data compared to those without it (16.7% vs. 14%, *p* = 0.003), likely due to a higher prevalence of SGA status in the HCA group (10.9% vs. 7.4%, *p* < 0.001) (Supplementary Table 3). Additionally, the proportion of infants excluded due to missing of ROP grading data was higher in the HCA cohort compared to the without-HCA cohort (11.0% vs. 13.6%, *p* < 0.001). These factors should be considered when interpreting the findings. Second, data on the degree of inflammation and the duration of fetal exposure to intrauterine inflammation were not collected from the KNN registry.

Nevertheless, this study is one of the largest to utilize a national registry to demonstrate a negative correlation between HCA and severe ROP in preterm infants, in line with findings from more recent studies. We also adjusted for important perinatal and neonatal factors associated with ROP development, such as GA, SGA, BPD, sepsis, and NEC.

In conclusion, after adjusting for confounding factors such as GA and birthweight, HCA was found to be negatively correlated with severe ROP in preterm infants. These findings are consistent with recent research, suggesting that HCA may offer a protective effect against severe ROP, potentially mediated by inflammatory markers. Despite the study’s limitations, the large study population and comprehensive adjustments strengthen the reliability of these findings.

## Supplementary information

Supplementary Information

## Data Availability

In line with the Korean Neonatal Network Publication Ethics Policy, all registered data are confidential and accessible only to researchers who have permission to access them for research activities. While the datasets generated and analyzed are not publicly available, they can be obtained from the corresponding author upon reasonable request.

## References

[1] Blencowe, H., Lawn, J. E., Vazquez, T., Fielder, A. & Gilbert, C. Preterm-associated visual impairment and estimates of retinopathy of prematurity at regional and global levels for 2010. *Pediatr. Res.* **74**, 35–49 (2013). 24366462 10.1038/pr.2013.205 PMC3873709

[2] Beligere, N. et al. Retinopathy of prematurity and neurodevelopmental disabilities in premature infants. *Semin Fetal Neonatal Med.* **20**, 346–353 (2015). 26235349 10.1016/j.siny.2015.06.004

[3] Ahn, J. H. et al. Neurodevelopmental outcomes in very low birthweight infants with retinopathy of prematurity in a nationwide cohort study. *Sci. Rep.* **12**, 5053 (2022). 35322163 10.1038/s41598-022-09053-8 PMC8943194

[4] Diggikar, S. et al. Retinopathy of prematurity and neurodevelopmental outcomes in preterm infants: a systematic review and meta-analysis. *Front Pediatr.* **11**, 1055813 (2023). 37009271 10.3389/fped.2023.1055813 PMC10050340

[5] Polam, S., Koons, A., Anwar, M., Shen-Schwarz, S. & Hegyi, T. Effect of chorioamnionitis on neurodevelopmental outcome in preterm infants. *Arch. Pediatr. Adolesc. Med.* **159**, 1032–1035 (2005). 16275792 10.1001/archpedi.159.11.1032

[6] Ludwig, C. A., Chen, T. A., Hernandez-Boussard, T., Moshfeghi, A. A. & Moshfeghi, D. M. The epidemiology of retinopathy of prematurity in the United States. *Ophthalmic Surg. Lasers Imaging Retin.* **48**, 553–562 (2017). 10.3928/23258160-20170630-06 28728176

[7] Bhatnagar, A., Skrehot, H. C., Bhatt, A., Herce, H. & Weng, C. Y. Epidemiology of retinopathy of prematurity in the US from 2003 to 2019. *JAMA Ophthalmol.* **141**, 479–485 (2023). 37052930 10.1001/jamaophthalmol.2023.0809 PMC10102919

[8] Villamor-Martinez, E. et al. Chorioamnionitis as a risk factor for retinopathy of prematurity: an updated systematic review and meta-analysis. *PLoS One* **13**, e0205838 (2018). 30332485 10.1371/journal.pone.0205838 PMC6192636

[9] Woods, J. & Biswas, S. Retinopathy of prematurity: from oxygen management to molecular manipulation. *Mol. Cell Pediatr.* **10**, 12 (2023). 37712996 10.1186/s40348-023-00163-5 PMC10504188

[10] Yucel, O. E., Eraydin, B., Niyaz, L. & Terzi, O. Incidence and risk factors for retinopathy of prematurity in premature, extremely low birth weight and extremely low gestational age infants. *BMC Ophthalmol.* **22**, 367 (2022). 36096834 10.1186/s12886-022-02591-9 PMC9469514

[11] Kim, S. J. et al. Retinopathy of prematurity: a review of risk factors and their clinical significance. *Surv. Ophthalmol. Surv. Ophthalmol.* **63**, 618–637 (2018). 29679617 10.1016/j.survophthal.2018.04.002 PMC6089661

[12] Dammann, O. et al. Immaturity, perinatal inflammation, and retinopathy of prematurity: a multi-hit hypothesis. *Early Hum. Dev. Early Hum. Dev.* **85**, 325–329 (2009). 19217727 10.1016/j.earlhumdev.2008.12.010

[13] Chen, M. L. et al. Placenta microbiology and histology and the risk for severe retinopathy of prematurity. *Investig. Ophthalmol. Vis. Sci.* **52**, 7052–7058 (2011). 21775664 10.1167/iovs.11-7380 PMC3207711

[14] Tsiartas, P. et al. The association between histological chorioamnionitis, funisitis and neonatal outcome in women with preterm prelabor rupture of membranes. *J. Matern Fetal Neonatal Med.* **26**, 1332–1336 (2013). 23489073 10.3109/14767058.2013.784741

[15] Lee, J. & Dammann, O. Perinatal infection, inflammation, and retinopathy of prematurity. *Semin Fetal Neonatal Med.* **17**, 26–29 (2012). 21903492 10.1016/j.siny.2011.08.007 PMC3242877

[16] Woo, S. J. et al. Effects of maternal and placental inflammation on retinopathy of prematurity. *Graefes Arch. Clin. Exp. Ophthalmol.* **250**, 915–923 (2012). 21455777 10.1007/s00417-011-1648-2

[17] Park, J. Y. et al. Retinopathy of prematurity in infants without fetal growth restriction is decreased with the progression of acute histologic chorioamnionitis: New observation as a protective factor against retinopathy of prematurity. *Placenta* **104**, 161–167 (2021). 33348284 10.1016/j.placenta.2020.12.007

[18] Athikarisamy, S. E., Lam, G. C., Cooper, M. N. & Strunk, T. Retinopathy of prematurity and placental histopathology findings: a retrospective cohort study. *Front. Pediatr.* **11**, 1099614 (2023). 36911032 10.3389/fped.2023.1099614 PMC9996070

[19] Owen, L. A. et al. Placental inflammation significantly correlates with reduced risk for retinopathy of prematurity. *Am. J. Pathol.* **193**, 1776–1788 (2023). 36822266 10.1016/j.ajpath.2023.02.003 PMC10616712

[20] International Committee for the Classification of Retinopathy of P The international classification of retinopathy of prematurity revisited. *Arch. Ophthalmol.* **123**, 991–999 (2005). 16009843 10.1001/archopht.123.7.991

[21] Fenton, T. R. & Kim, J. H. A systematic review and meta-analysis to revise the Fenton growth chart for preterm infants. *BMC Pediatr.* **13**, 59 (2013). 23601190 10.1186/1471-2431-13-59 PMC3637477

[22] Bell, M. J. et al. Neonatal necrotizing enterocolitis. Therapeutic decisions based upon clinical staging. *Ann. Surg.* **187**, 1–7 (1978). 413500 10.1097/00000658-197801000-00001 PMC1396409

[23] Papile, L. A., Munsick-Bruno, G. & Schaefer, A. Relationship of cerebral intraventricular hemorrhage and early childhood neurologic handicaps. *J. Pediatr.* **103**, 273–277 (1983). 6875724 10.1016/s0022-3476(83)80366-7

[24] Stoll, B. J. et al. Late-onset sepsis in very low birth weight neonates: the experience of the NICHD Neonatal Research Network. *Pediatrics* **110**, 285–291 (2002). 12165580 10.1542/peds.110.2.285

[25] Ehrenkranz, R. A. et al. Validation of the National Institutes of Health consensus definition of bronchopulmonary dysplasia. *Pediatrics* **116**, 1353–1360 (2005). Pediatrics. 16322158 10.1542/peds.2005-0249

[26] Moscuzza, F. et al. Correlation between placental histopathology and fetal/neonatal outcome: chorioamnionitis and funisitis are associated to intraventricular haemorrage and retinopathy of prematurity in preterm newborns. *Gynecol. Endocrinol.* **27**, 319–323 (2011). 20528214 10.3109/09513590.2010.487619

[27] Seliga-Siwecka, J. P. & Kornacka, M. K. Neonatal outcome of preterm infants born to mothers with abnormal genital tract colonisation and chorioamnionitis: a cohort study. *Early Hum. Dev.* **89**, 271–275 (2013). 23158015 10.1016/j.earlhumdev.2012.10.003

[28] Ahn, Y. J. et al. Characteristic clinical features associated with aggressive posterior retinopathy of prematurity. *Eye* **31**, 924–930 (2017). 28234354 10.1038/eye.2017.18 PMC5518835

[29] Kim, S. Y. et al. Neonatal morbidities associated with histologic chorioamnionitis defined based on the site and extent of inflammation in very low birth weight infants. *J. Korean Med. Sci.* **30**, 1476–1482 (2015). 26425046 10.3346/jkms.2015.30.10.1476 PMC4575938

[30] Mitra, S., Aune, D., Speer, C. P. & Saugstad, O. D. Chorioamnionitis as a risk factor for retinopathy of prematurity: a systematic review and meta-analysis. *Neonatology* **105**, 189–199 (2014). 24481268 10.1159/000357556

[31] Lee, S. E., Han, B. D., Park, I. S., Romero, R. & Yoon, B. H. Evidence supporting proteolytic cleavage of insulin-like growth factor binding protein-1 (IGFBP-1) protein in amniotic fluid. *J. Perinat. Med.* **36**, 316–323 (2008). 18598121 10.1515/JPM.2008.067 PMC3162369

[32] Bae, J. H., Song, D. K. & Im, S. S. Regulation of IGFBP-1 in metabolic diseases. *J. Lifestyle Med.* **3**, 73–79 (2013). 26064841 PMC4390745

[33] Siddals, K. W., Westwood, M., Gibson, J. M. & White, A. IGF-binding protein-1 inhibits IGF effects on adipocyte function: implications for insulin-like actions at the adipocyte. *J. Endocrinol.* **174**, 289–297 (2002). 12176668 10.1677/joe.0.1740289

