# Supplementary material for: Correlation between histologic chorioamnionitis and severe retinopathy of prematurity
Source: Pediatr Res. 2025 May 16;98(6):2139–43. doi: 10.1038/s41390-025-04093-y (PMC12811112; doi:10.1038/s41390-025-04093-y)
Supplement: Supplementary file 1 — Supplementary Information [file 41390_2025_4093_MOESM1_ESM.pdf]

Supplementary Tables

Supplementary Table 1. Duration of respiratory support according to severe retinopathy of prematurity in each cohort.

Supplementary Table 2. Demographics findings and neonatal outcomes according to the gestational age stratification.

Supplementary Table 3. Characteristics of infants with HCA data and without HCA data.

Supplementary Table 1. Duration of respiratory support according to severe retinopathy of prematurity in each cohort

	Without-HCA			HCA		
	no ROP	ROP	*p*-value	no ROP	ROP	*p*-value
Duration of invasive ventilation (days)	10.1±19.4	40.8±37.8	<0.001	13.4±26.4	47.2±42.2	<0.001
Duration of non-invasive ventilation (days)	22.3±19.9	36.6±26	<0.001	25.6±21.2	39.7±28	<0.001
Duration of supplementary oxygen (days)	6.5±11.4	13.2±17.6	<0.001	7.8±12.8	14.9±19	<0.001

Values are expressed as means ± standard deviations (SDs).

HCA, histologic chorioamnionitis; ROP, retinopathy of prematurity.

Supplementary Table 2. Demographics findings and neonatal outcomes according to the gestational age stratification

	GA 23–25 weeks (n=1,879)	GA 26–28 weeks (n=4,979)	GA 28–31 weeks (n=5,274)	*p*-value
GA (week)	24.8±0.8	27.6±0.9	30.2±0.8	<0.001
Birthweight (gram)	727.2±133.9	1017.9±209.3	1242.7±201.2	<0.001
Birthweight (z-score)	0.2±0.9	0.1±0.8	-0.4±0.7	<0.001
SGA	116 (6.2)	369 (7.4)	732 (13.9)	<0.001
Cesarean section	1334 (71)	3949 (79.3)	4378 (83)	<0.001
Female	911 (48.5)	2361 (47.5)	2717 (51.5)	<0.001
Oligohydramnios	270 (15.5)	680 (14.6)	618 (12.4)	<0.001
Prenatal steroid	1625 (87.1)	4371 (88.6)	4596 (87.7)	0.157
Multiple birth	665 (35.4)	1622 (32.6)	2146 (40.7)	<0.001
PPROM	883 (47.2)	1956 (39.5)	1825 (34.7)	<0.001
HCA	1048 (55.7)	1995 (40.1)	1353 (25.7)	<0.001
Neonatal outcomes				
RDS	1849 (98.4)	4648 (93.4)	3743 (71)	<0.001
NEC	272 (14.5)	330 (6.6)	150 (2.8)	<0.001
IVH ≥ Gr 3	1318 (70.2)	2221 (44.6)	1583 (30)	<0.001
Sepsis	729 (38.8)	1158 (23.3)	516 (9.8)	<0.001
Moderate to severe BPD	1346 (75.2)	2101 (43)	1008 (19.2)	<0.001
Severe ROP	1116 (59.4)	836 (16.8)	129 (2.5)	<0.001
Death	99 (5.3)	95 (1.9)	25 (0.5)	<0.001

Values are expressed as N (%) or means ± standard deviations (SDs).

GA, gestational week; SGA, small for gestational age; PPROM, preterm premature rupture of membrane; HCA, histologic chorioamnionitis; RDS, respiratory distress syndrome; NEC, necrotizing enterocolitis; IVH, intraventricular hemorrhage; BPD, bronchopulmonary dysplasia; ROP, retinopathy of prematurity.

Supplementary Table 3. Characteristics of infants with HCA data and without HCA data

	Infants with HCA data (n=13,778)	Infants without HCA data (n=2,278)	*p*-value
GA (week)	28±2.3	28.1±2.3	0.119
Birthweight (gram)	1036.5±282.2	1061±274	<0.001
SGA	1506 (10.9)	168 (7.4)	<0.001
Female	6723 (48.8)	1146 (50.3)	0.189
RDS	11789 (85.6)	2052 (90.1)	0.118
NEC	991 (7.3)	179 (8)	0.239
Sepsis	2831 (20.6)	494 (21.8)	0.209
Moderate to severe BPD	4495 (37.3)	614 (32.5)	0.366
Severe ROP	2081 (16.7)	281 (14)	0.003

Values are expressed as N (%) or means ± standard deviations (SDs).

HCA, histologic chorioamnionitis; GA, gestational age; SGA, small for gestational age; RDS, respiratory distress syndrome; NEC, necrotizing enterocolitis; BPD, bronchopulmonary dysplasia; ROP, retinopathy of prematurity
